# Egg Cryopreservation for Social Reasons—A Literature Review

**DOI:** 10.3390/healthcare12232421

**Published:** 2024-12-02

**Authors:** Stavroula Kynigopoulou, Alkis Matsas, Ermioni Tsarna, Smaragdi Christopoulou, Periklis Panagopoulos, Panagiotis Bakas, Panagiotis Christopoulos

**Affiliations:** 1 MSc Program “Research in Female Reproduction”, Medical School, National and Kapodistrian University of Athens, 115 28 Athens, Greece; 2 Laboratory of Experimental Surgery and Surgical Research ‘N.S. Christeas’, Medical School, National and Kapodistrian University of Athens, 115 27 Athens, Greece; 3 Second Department of Obstetrics and Gynecology, “Aretaieion” Hospital, Medical School, National and Kapodistrian University of Athens, 115 28 Athens, Greece; 4 Third Department of Obstetrics and Gynecology, University Hospital “ATTIKON”, Medical School, National and Kapodistrian University of Athens, 124 62 Athens, Greece

**Keywords:** social egg freezing, oocyte cryopreservation, egg cryopreservation, social reasons

## Abstract

This paper provides an overview of the social reasons that lead women to consider egg cryopreservation, as well as the attitudes and knowledge of women towards this procedure. Methods: For the creation of this article, a literature review was carried out both in the existing medical literature and in search engines. The key points are as follows: (1) The main reasons women choose to delay childbearing are the lack of a suitable male partner, education and career advancement, and financial instability. Women feel societal and family pressure to have children, even though they may not feel ready. (2) Women’s attitudes towards egg cryopreservation vary. While some are open to the idea, many are opposed to it, especially when it is used for non-medical reasons. There are concerns about the success rates, health risks, and ethical implications of the procedure. (3) The legal and regulatory framework around egg cryopreservation differs across countries. Some countries allow it only for medical reasons, while others have more permissive policies. The issue of who should bear the financial cost of the procedure is also debated. (4) There is a need to better educate women, as well as healthcare providers, about fertility decline and the options available for preserving fertility, including egg cryopreservation.

## 1. Introduction

It is known that the fecundity of women decreases gradually over the years. The decrease starts at the age of 32 and rapidly changes after the age of 37 [1]. An ever-increasing number of women choose to delay childbearing for various reasons. Assisted Reproductive Technology (ART) has evolved with time due to the constantly increasing need to preserve female fertility. Intracytoplasmic sperm injection (ICSI), oocyte, sperm, and embryo cryopreservation, and in vitro fertilization (IVF) list the different ART methods. ART has been around for over 40 years providing both physicians and patients with ever evolving techniques. Embryo and sperm cryopreservation are already widely considered ART-effective techniques. Oocyte cryopreservation nowadays tends to be more in the spotlight. Due to its improved techniques regarding safety and efficiency, both the Practice Committees of the American Society for Assisted Reproductive Technology and the European Society of Human Reproduction and Embryology (ESHRE) Task Force on Ethics and Law do not consider oocyte cryopreservation to be experimental anymore [2,3]. As a result, it is already considered the method of choice for preserving female fertility not only by women who face medical problems and conditions but also by women who are considered physically healthy, thus providing the opportunity for these women to have biological offspring and become mothers while avoiding making neither haste nor important decisions right on the spot [4].

The use of said method for non-medical reasons raises various ethical, social, economic, and legal questions. The purpose of this paper is to review the various social reasons that may or may not interfere with women’s decision-making concerning egg freezing collected from articles.

### 1.1. Brief History Report

Fertility has always been at the center of worshiped rituals by all kinds of different cultures throughout the existence of mankind since ancient times all over the world. Museums all over the world present artifacts such as little or big stone statues, paintings, and various types of metallic creations dedicated to deities associated with the stimulation of production either in humans or in the natural world. Africans, ancient Egyptians, Incas, Inuits, Aztecs, Mayans, Chinese, Indians, Celtics, Germanic, ancient Greeks, Romans, Slavics, and Indigenous Australians are but a few of the well-studied civilizations that had their own list of fertility gods and goddesses to pray to. Furthermore, when Ancient Greek physicians were facing reproductive failure in married couples, procreative disruption took place and thus “infertility” was noted. If after a long list of medical either liquid or surgical remedies the problem of childlessness remained, there were gods upon who ancient Greeks would pray to resolve the problem [5]. Therefore, fertility in both the human and the natural world and its celebration goes back centuries and has a long history.

### 1.2. Definition of ART [6]

Assisted reproductive technologies (ART) by the American Center for Disease Control (CDC) are fertility-related treatments that help in achieving pregnancy conception in individuals who are having difficulty doing so spontaneously. They are procedures during which oocytes or embryos are manipulated. Any procedure that involves sperm manipulation is not included in this definition. Procedures that involve ovarian stimulation without retrieving an egg in the end, are also excluded from said definition. In vitro fertilization (IVF) is by far the most common ART procedure preferred alongside cryopreservation and intracytoplasmic sperm injection (ICSI).

### 1.3. Definition of Cryopreservation

Cryopreservation is the technique during which any type of biological construct is preserved for any amount of time, by being cooled at a very low temperature [7]. Physically there is water inside the cells and there is water surrounding them. Therefore, when we try to freeze living cells or tissue, we submerge the sample in an extremely cold liquid, resulting in ice formation both inside the cell and in its surrounding environment. This causes changes in the cellular structure and then damage. Cryoprotective agents are used to help overcome the obstacles of the cells of water-to-ice transition. These liquids were developed to minimize the ice formation both inside and outside of the cells no matter how low the temperatures.

**Figure 1:**
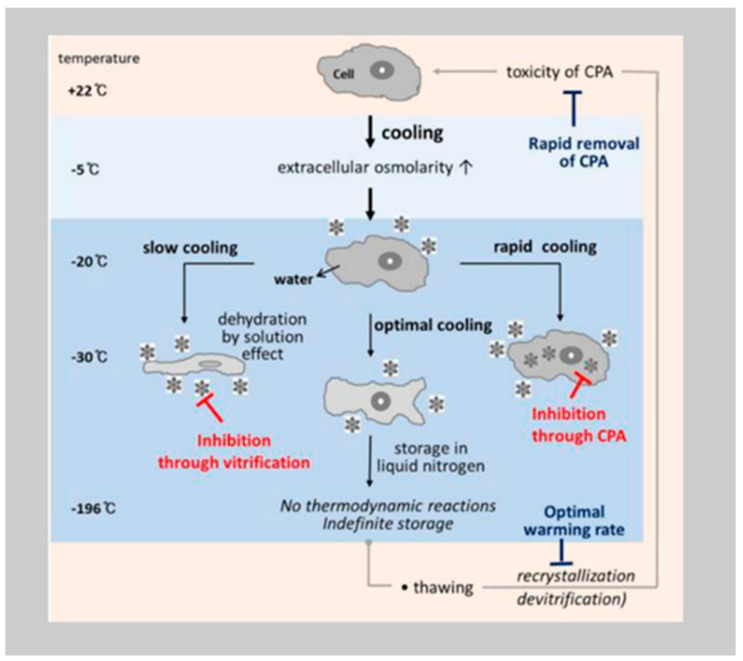
Physical events and cryoinjury of cells during freezing and thawing. Cryoinjuries are caused, at least in part, by the solution effect (leading to osmotic shock) and intracellular ice formation (leading to the breakdown of intracellular structures) [7].

The following figure demonstrates the events of cellular cryoinjury (Figure 1).

### 1.4. IVF and Cryopreservation Law

Assisted reproductive techniques have had their definition originally established by Greek law in late 1999 with surrogacy being the first term to be legislated. Heterosexual couples that were trying to have a child outside of wedlock had a lot of legal things to deal with. Mutual consent and recognition of the offspring were being individualized in every case due to at that time insufficient Greek legislation. The Greek legal framework concerning assisted reproduction was originally established in 2002 and regulated kinship and inheritance rights for children who were born with the help of assisted reproduction. According to article 1455 of Greek law 3089/2002, assisted reproduction was only allowed in case a couple dealt with infertility, or they were trying to avoid passing on to the child some kind of serious illness. The couple did not have the right to predetermine the sex of the baby unless a severe sex-related inheritable disease was at stake. It had also been regulated by the same law, the transfer of fertilized eggs without compensation.

After only 3 years, a new legal framework was established. Even though assisted reproduction methods were increasingly wildly spreading in the private medical community, there had been a clarification neither for the exact assisted reproduction techniques that were being performed nor for the proper operation of private assisted reproduction clinics or the cryopreservation banks. Women’s age limit for undergoing assisted reproduction was set to be their 50th year and the time allowed to cryopreserve semen and testicular tissue, embryos, unfertilized oocytes, and ovarian tissue was 10 years, 5 years (with a 5-year time extension), and 5 years accordingly. Penalties for failure to comply with the conditions set by the law were also regulated. That newly established law still stands to this day with some alterations. According to the recent Greek legislation 4958/2022, the women’s age limit for undergoing assisted reproduction was reformed to 54 years. In addition, the law states that gametes or embryo cryopreservation apply for both therapeutic and fertility preservation purposes. In any case of gamete or embryo cryopreservation, the time of extensions has been set to be indefinite [8].

According to the European Society of Human Reproduction and Embryology [9] and its following tables (Table 1 and Table 2), legal legislation regarding oocyte cryopreservation varies across the countries in Europe. In 2017, some European countries had specific regulations concerning oocyte cryopreservation, but none of that resulted in any kind of funding or even a partial refund for social egg freezers. Fourteen out of twenty-seven countries allow egg freezing when there is a pre-existing medical reason, whereas, in Austria, France, and Malta, social egg freezing is forbidden. The rest of the countries in Europe continue to practice oocyte freezing regardless of the lack of a specific legal regime. Estonia and Lithuania are the only two European countries that have no regulations, no indications, and no funding for oocyte cryopreservation, while Ireland funds women who undergo egg freezing for medical reasons. Belgium, Denmark, France, Germany, Greece (according to the newly reformed law), Malta, and Slovenia have set a time limit to allow women to cryopreserve their eggs, whereas Spain just requires women to be adults.

The 2021 European Atlas of Fertility Treatment Policies assesses assisted reproduction regulations across Europe, highlighting the differences in access and funding. Belgium, Israel, and the Netherlands lead with the highest access scores (86%), providing comprehensive fertility treatments and public funding. Moderate access is seen in Portugal, Finland, and Norway, while Greece, Spain, and Italy face restrictions despite public funding. Countries such as Albania, Poland, and Ireland offer little support, burdening citizens financially. The atlas recommends harmonizing legislation, increasing public funding, and launching awareness campaigns to improve access and reduce the stigma surrounding fertility treatments [10].

#### 1.4.1. Cryopreservation Law in Asia

According to the current Chinese regulations, specific groups of people that do not meet the criteria about national population and family planning are not allowed to be artificially reproductively assisted [11]. These groups include single or unmarried women, unmarried couples, homosexuals, transgender people, as well as people performing high-risk occupations [12]. Although only the Chinese Jilin province may have made a regulative exception about ART concerning the previously mentioned groups, local physicians support that said exception has never been put into practice because the local hospitals do not want to go against the national health authority [13]. In Japan [14], women with pre-existing pathological reasons affecting future fertility are allowed to undergo oocyte or oocyte tissue cryopreservation only after they are appropriately and rightfully informed about the procedure and its possible effects. Women who wish to avoid age-related infertility are also legally allowed to take refuge in cryopreserving their eggs according to the Japanese Ethics Committee of Reproductive Medicine. According to Russian federal laws [15], every woman eligible for childbearing has the right to access any ART. Although up until recently Russian clinics would refuse to treat single men or women to surrogacy via artificial fertilization, due to the issues that would arise for the children that would be born from surrogacy to a single-parent family, thanks to new Russian law, that has started to shift. There have been several cases of single parents (regardless of their sex) who were registered by Russian courts as single parents to their adoptive child (via surrogacy), proving that even though Russia could be considered a reproductive paradise due to its low ART practice costs, the one thing that has not changed through time is that the only indications for women to seek help in ART that are justified by the Russian law are medical ones.

#### 1.4.2. Cryopreservation Law in USA, Australia, Canada, and UK

In the USA [16], the legal framework for ART and egg cryopreservation is a little different than that of the rest of the world. Sperm, egg, and embryo cryopreservation are under the protection of US federal laws, and single and married women are allowed to hire surrogate parents. Unfortunately, no other regulations, including that of embryo disposal, are set by each case and court in individual states. We come across similar constitutional laws both in Australia and Canada as well [17]. There is a basic legal framework that embodies ART and surrogacy; however, every case is different, and every state can regulate its own legal regime. Women who have been declared infertile or have a higher chance of having a child with a hereditary disease (married, single, or lesbian) have the right to access any form of ART. Little is classified, though, about single women trying to undergo egg cryopreservation to avoid age-related infertility, same-sex couples, and transgender people when resulting in any form of ART.

**Figure 2:**
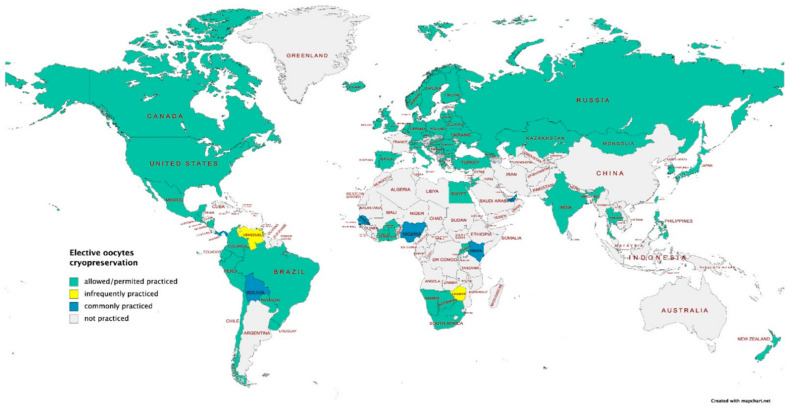
Worldwide map of practiced SEF [18].

**Figure 3:**
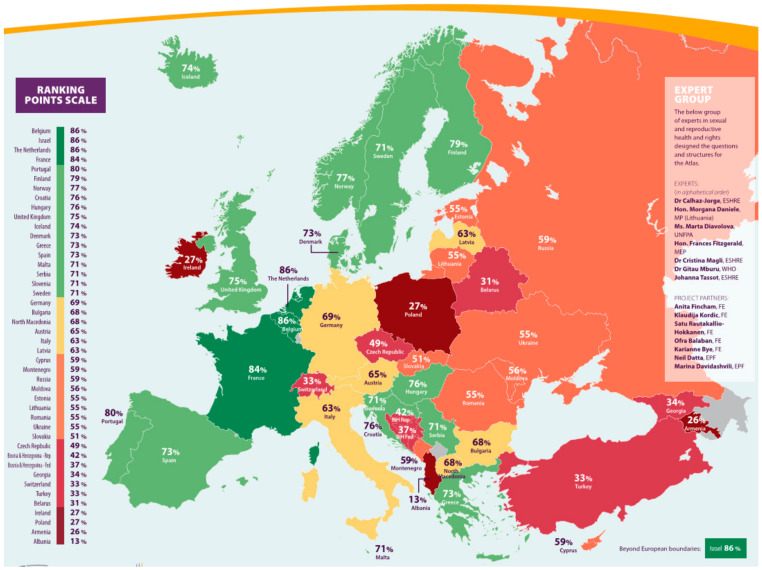
European Atlas of Fertility Treatment Policies [10].

According to Varlas et al. [18] and their paper in 2021, the following image depicts the countries in which Selective Egg Freezing (SEF) is permitted or practiced (countries with the green color), the countries in which it is a common practice (blue depicted countries), and the countries in which is not a common practice (yellow depicted countries), while in grey are depicted countries in which SEF is not practiced at all (Figure 2). According to Figure 3, the main findings on Fertility Treatment Policies in Europe and the analysis of access to assisted reproduction methods reveal significant disparities between countries. Belgium, Israel, and the Netherlands lead with the highest access scores (86%), providing comprehensive fertility treatments such as IVF and ICSI, along with significant public funding. Portugal (80%), Finland (79%), and Norway (77%) offer moderate access with partial funding and some restrictions. In contrast, Greece (73%), Spain (73%), and Italy (63%) have limited access due to geographical and economic differences, while Albania (13%), Poland (27%), and Ireland (27%) exhibit lower access with little public support, placing a financial burden on citizens seeking treatments [10].

### 1.5. IVF and ICSI

In vitro fertilization (IVF) is a method during which both sperm and eggs are added together in a dish to be fertilized whereas ICSI involves injecting a single sperm into a single egg [19]. ICSI is highly effective when dealing with male factor infertility [19], and there are currently various indications for ICSI that are widely adopted, rendering it the most popular insemination method worldwide [20]. In both methods, when those embryos are successfully fertilized and matured, either they are inserted in the women undergoing the infertility treatment right away or they are cryopreserved, and a new later date is set for the embryo implantation.

### 1.6. Cryopreservation Techniques

Cryopreservation involves the preservation of cells and tissue for long periods of time at sub-zero temperatures. Oocyte cryopreservation is an established and widely used treatment for women who are willing to postpone childbearing. As a procedure, oocyte cryopreservation involves ovarian hormonal stimulation, oocyte retrieval, freezing, and oocyte storage [21]. Cryoprotective additives (CPAs) are used to reduce cryodamage by preventing ice formation. There are two basic techniques applied to the cryopreservation of human oocytes: controlled slow freezing, which was more commonly used in the early years, and ultrarapid cooling by vitrification, which is more commonly used nowadays. Slow freezing results in a liquid changing to a solid state, whereas vitrification results in a non-crystalline amorphous solid [22]. According to Argyle et al. in a systematic review they conducted in 2016, vitrification is the cryopreservation technique of choice [22]. Embryos can also be cryopreserved and are widely used in IVF treatments but that requires legal ownership between both partners, which is more complex and leads to further difficulties. The European Society of Human Reproduction and Embryology states that, for example, if a couple has cryopreserved their embryos but ends up splitting prior to the IVF treatment, the person willing to become pregnant cannot do so if their partner has retracted their consent [23].

#### 1.6.1. Optimal Timing of Cryopreservation

It is well known that the success rates of IVF cycles using cryopreserved oocytes decline rapidly as the age of women increases [24]. The optimal time for women to freeze their eggs, with a much higher success rate, is under 35 years according to systematic studies from both Alteri et al. and Varlas et al. [18,25]. Cryopreserving eggs at an earlier age can minimize the number of cycles necessary to obtain sufficient eggs and maximize egg quality, with the risk of never using them [18]. In women aged ≤35 years, the cumulative live birth rate (CLBR), considering the total number of oocytes used in consecutive procedures, was significantly higher compared with those aged >36 years, despite the same number of oocytes being utilized [25,26]. Another survey by Doyle et al. came to a similar conclusion that to achieve the highest probability of live birth rate from cryopreserved oocytes, these oocytes should have been retrieved prior to the age of 36–38 years [27]. Unfortunately, most women undergo treatment after a significant decline in fertility. Indeed, studies based on surveys have shown that the average age of cryopreserve oocytes is between 36 and 38 years [25].

#### 1.6.2. The Optimal Number of Oocytes

The number of oocytes retrieved should be individualized according to each patient’s age, ovarian reserve, and clinical circumstances. Different studies about fertility preservation from all over the world lead up to the same conclusion, that the better the quality of the oocyte used, the higher the success rate of a healthy pregnancy outcome and a viable genetically related offspring. According to Doyle et al.’s study, women between 35 and 38 years of age with 20 cryopreserved oocytes would have live birth rates of 80% and 60%, respectively [27]. Meanwhile, Cobo et al. concluded that pregnancy rates are age-dependent and a minimum of 8–10 oocytes need to be retrieved to achieve pregnancy [28]. Cryopreserving a small number of oocytes can lead to low success pregnancy rates.

#### 1.6.3. Imagistic Diagnosis (Ultrasound Findings) Correlated with the Ovarian Cancer Markers

The diagnosis of ovarian cancer using ultrasound and cancer markers has emerged as an important tool for the detection and assessment of the disease. According to Yang et al. [29], the combination of morphological features determined by ultrasound and the measurement of tumor markers, such as CA-125 and HE4, can significantly increase accuracy in the early detection of ovarian cancer. The evaluation of ultrasound images through the Doppler technique, which enhances the visualization of blood vessels, is now considered essential in cases of suspicious findings, as malignant tumors show increased perfusion and low resistance [30]. In addition, the application of markers, such as HE4 and Risk of Ovarian Malignancy Algorithm (ROMA), appears to offer a significant advantage in prognostic accuracy, especially when used in conjunction with ultrasound findings [29]. The literature review demonstrates that the use of CA-125 as a marker may be more effective when evaluated alongside the morphological elements of the ultrasound. Recent studies demonstrate that this combination can increase the sensitivity of the diagnosis while also offering possibilities for more accurate staging of the disease [29,30]. This diagnostic approach enhances the ability for early detection and targeted management of the disease, offering better treatment and survival prospects for ovarian cancer patients.

## 2. Materials and Methods

### 2.1. The Purpose of This Paper

The purpose of this paper is to review the reasons women of reproductive age, free from pathological medical conditions, would turn to cryopreserving their oocytes to prolong fertility and postpone pregnancy. For the creation of this article, a literature review was carried out both in the existing medical literature and in search engines. For that reason, the use of PRISMA criteria was not followed. Nevertheless, for the development of the literature review, a methodical search was conducted using well-known databases such as Google Scholar (1980–2023), PubMed (1980–2023), Scopus (1980–2023), Medline (1980–2023), and Elsevier (1980–2023). Results related to the present topic were identified, and then they were reviewed and included in the Results Section of this scientific article.

### 2.2. Methods

An online search of databases was performed using Google Scholar (1980–2023), PubMed (1980–2023), Scopus (1980–2023), Medline (1980–2023), and Elsevier (1980–2023). The combination of words that were used for citations was for oocyte cryopreservation (“oocyte cryopreservation”, “oocyte freezing”, “egg freezing” or “egg cryopreservation”) and social reasons (“social reason*”). To generate final citations on all databases ‘AND’ was used between oocyte cryopreservation and social reasons. The filter for citations regarding human studies was also set. Language restrictions were used on the search so that only English-published articles could be included. Exclusion criteria were citations that either would provide only the abstract or there was no free way to access their results. Citations that were also not included in this paper were studies that oocyte cryopreservation was the only fertility preservation option due to any form of pre-existing pathological or oncological condition (Table 3).

## 3. Results

### 3.1. Social Reasons Lead Women to Egg Cryopreservation and Women’s Attitude Regarding ART

Oocyte cryopreservation has technologically improved in the last 30 years, and it is no longer considered only as a fertility treatment for cancer patients or for women with severe medical conditions, but also as a solution for women dealing with age-related fertility loss. Oocyte cryopreservation or egg freezing for non-medical reasons is increasing and has, to a certain point, replaced embryo cryopreservation, according to ESHRE, for women without a male partner to preserve their fertility. Women undergoing egg cryopreservation are usually healthy and do not have a pre-existing medical condition that would endanger their ability to become mothers in the future. Therefore, the motivations, in this case, are a little different [23]. A recent Royal College of Obstetricians and Gynaecologists (RCOG) scientific opinion piece on the topic stated that elective egg freezing for non-medical reasons provides an opportunity for women to mitigate the decline in their fertility with age but highlights that women undertaking oocyte cryopreservation should only do so with a full understanding of the likelihood of success [21]. Various studies worldwide have investigated the knowledge, attitude, and awareness women have concerning fertility decline and egg freezing. They all conclude that women need to be better and fully informed about the optimal time of having a child as well as that further investigation is needed about the always-evolving reproductive technological advancements [14,31,32].

#### 3.1.1. Lack of Partner

In a study conducted in the Netherlands [33], when women were questioned about their motivations as to why they were trying egg freezing, two reasons came up most often: rapid fertility decline and lack of a male partner. When women felt the pressure of time on starting a family and realized that their fecundity was deteriorating, it was realized that the only way to fulfill the desire to have a baby was with the medical aid of egg cryopreservation. The constant stress of a medical ‘timetable’ led to various psychological issues. Some women even experienced health problems such as depression and fatigue due to the constant denial of either a partnerless or a childless future. Thus, it was considered by women, discussing future family plans with relatives and friends, an inappropriate matter [33]. When women talked about cryopreserving their eggs, a sense of ‘breathing space’ was created. When those women would consider their eggs “being in the freezer”, the idea of insurance was revived, and a weight was lifted off their shoulders. Even though it was understood that their frozen eggs were not a guarantee of a future living offspring, it was believed that an effort should be made to achieve that. Not taking any action most often would lead to regret. Hope about the future and peace of mind were the results of ‘the backup plan’ [33].

When women were questioned about their future family ‘picture’, the most significant aspect was a same-minded partner and a stable relationship. It was vital for women for the family to consist of a father and a mother raising a genetically related child. Things women wished for included the child to bear similar physical characteristics, passed on from its parent’s genes. Women coming from all types of family backgrounds (either small or big families) realize the importance of family. Even women who might have had doubts about having children would prefer to have a time limit when starting to create a family. Even though most women would prefer, when asked, to have a baby in the “natural” way, when time started to pass, they too started to consider alternative options and ways of becoming mothers. Other ways were single motherhood or even having children and co-parenting with male homosexual couples. There were also women who expressed their doubts about taking on single motherhood due to the excess financial responsibility they would have to take. Some women would also give up the ideal look of a family and end up considering adoption or fostering children as well [33].

A study conducted in Spain [14] also revealed that over 70% of single women’s motivation for undergoing IVF/ICSI and more than 60% that electively froze their egg was, in both cases, due to the lack of a partner. An interesting finding in the study was that women who underwent IVF/ICSI perceived stronger family support than women undergoing SEF. Another conclusion of the study was that all women who underwent SEF knew the possibility of needing a sperm donor, whereas only 59.9% of women undergoing IVF/ICSI were informed about SEF. The results of said study, in contradiction with the one conducted in the Netherlands [33], showed the importance of family ties during women’s decision-making when and if starting IVF/ICSI as single women and the misinformation of women about SEF as an established method of delayed motherhood.

Jones et al. study [34] in the UK came to a similar conclusion that 70% of women who participated in freezing their eggs were influenced by the lack of a partner. In a retrospective analysis in the UK [35] that succeeded Jones’s study [34], it was also noted that women’s motivations for undergoing egg freezing in the first place was also the lack of a male partner. The British analysis resulted in the singleness of all women who underwent egg freezing when returned to use their originally frozen eggs.

In Belgium [36], Pennings, in 2021, wanted to look deeper into the actual reason for the lack of partners for highly educated women and he concluded that women were concerned about “marrying down” if they chose a partner with a lower degree in education. For decades, it was stereotypically expected by people that the major role a person had to play was that of a parent. Through marriage, heterosexual couples were expected to have biologically related children, while the male, because of his more advanced education than his partner, would earn more and be the breadwinner, and the woman would be the homemaker. But after decades of constant efforts, the number of women trying and achieving tertiary education has increased, thus leading to an inevitable gender inequality. Relationships were created where both male and female counterparts felt that they were being wronged. Some men were intimidated by the fact that their partners had either a higher degree in education than them or were earning more, or sometimes even both, whereas women have had to put up with a lot of pressure when dealing with the economic and educational advantages men receive that now it is hard for them to settle for something less. Hence, the reversed gender mismatch was cultivated. Egg freezing has resulted in some sort of reproductive autonomy for women; however, the problem of social imbalance between the two genders remains. Although the ever-advancing technology has procured more safety for both sexes and their gametes, Pennings’ study resulted in the irreversibility of the gender education gap.

#### 3.1.2. Education and Career Advancement

Among the reasons women are considering delaying childbearing are also education and career prioritization [37]. It is a noticeable fact that, worldwide, women have been trying to extend their knowledge and ascend in the workplace. When female residents in the USA [38] were questioned about the reasons leading to postponing pregnancy, residency was considered of vital importance by more than 70%. Both career plans and future childcare were also considered important reasons to delay the creation of a family by more than 50% of the female residents. SEF being very attractive to people with higher education was also confirmed in Schick’s study [39]. More participants in said German study with a university degree understood the use of SEF due to the unwillingness to balance both a career and a family.

#### 3.1.3. Financial Instability and SEF

When women were questioned about the financial journey, they would take upon cryopreserving their eggs, it was concluded that said reproductive technique should be offered to everybody, regardless of their income. It is considered by many women that if SEF was to be more widely financially accessible, more women would be given the opportunity to plan their future regarding childbearing [40]. Women also noted the aid of their drastic income increase led to easier access to egg freezing. The discrimination between women who can afford to pay to have their eggs frozen and those who cannot was inevitable. There is also another issue that was unveiled. When women were informed that either homosexual couples or heterosexual couples battling with long-life infertility would receive financial coverage, they felt discriminated against. The idea of creating the same opportunities for all remained. But having single mothers bear the financial burden on their own was deemed unfair. That led to a suggestion that cryopreservation expenses be covered by either healthcare insurance or the state [33,41]. Partial compensation was also considered a viable option by many [33].

The economic burden of SEF is not only on the minds of people undergoing it, but it is also a worldwide concern. Both international company employers [42] and bioethicists [43,44] have been in a constant battle as to whether full or even partial coverage for SEF should be offered and by whom. There is an ongoing question as to who deserves to be compensated for SEF procedures, why, and how much the right amount of compensation is. Every woman who decides to go down that path has different characteristics and needs. There are women who have already had a pre-existing medical condition that prevented them from getting pregnant naturally on their own, older women whose fertility is declining rapidly, women who simply lack a male partner, and women who choose to postpone childbearing for some time. Therefore, the need for compensation should be differentiated in every single case. Egg cryopreservation and its individual procedures (ovarian stimulation, oocyte retrieval, cryopreservation, storage, and later, if necessary, thawing and fertilization) are considered a well-established method today for countereffect infertility. In most countries, infertility treatments are financially covered by the public healthcare system. Therefore, the logical thing would be for each country’s public healthcare system to cover any expenses on SEF. But the reality is far from that. Private fertility clinics offer their services; therefore, most women and couples end up covering their own expenses with no refund at all.

On the one hand, according to bioethicists [44,45], corporations attempting to entice people and benefit their employees with infertility procedure coverage contracts were perceived poorly. Companies were suggested to find other ways to show their family-friendly policies. Increased benefits, longer maternal and/or paternal work leave, or even assistance with daycare were some of the new policies that were suggested. On the other hand, other bioethicists [46] argued with the belief that company-sponsored egg freezing limits women’s autonomous choice about family making. Under well-informed and specific conditions, company policy guidelines, and no pressure, women should ensure their autonomous choices about the time they would choose to start their family. It was also pointed out that if any woman felt the need to comply with their employer’s dictations no matter their own personal beliefs and wishes, this was no different than any other type of pressure the labor market would put on them that would be more easily accepted.

The ethical dimension of oocyte cryopreservation (OoC) technology provokes various debates about its potential implications for individuals and society. From the individual’s point of view, the issues that arise relate to the physical and psychological burden, the dimension of the choice to reproduce, and the potential exploitation of the right to reproduce. From a social perspective, this technology may affect traditional notions of family and parenthood while raising questions about equality in access to these technologies [47].

### 3.2. Women’s Attitude on SEF

A recent study in Italy by Tozzo et al. asked 930 female students of Padova University about their understanding and attitude towards social egg freezing [48]. Data collected in this study revealed some important points about young women and their knowledge about social oocyte freezing in Italy compared to other European countries and the United States. Overall, 34.3% of the students reported having heard about the possibility of oocyte cryopreservation for non-medical reasons and being aware of the meaning of this procedure; only 19.5% were in favor of social egg freezing, and 48.4% thought that the cost for this procedure should be borne entirely by the woman herself. The study showed that young Italian women are significantly less aware of the age-related decline in fertility and the possibility of using social egg freezing compared to their similarly situated counterparts in other Western countries.

In Austria [49], when women were asked about their opinions and viewpoints concerning egg freezing, interesting results were identified. Despite Austria being one of the countries in Europe in which egg-freezing is not allowed—neither for medical nor for social reasons—and even though women believe that having a child is not just a ‘right’, it was highly perceived that women should be able to decide for themselves regardless of the different types of policy interventions. In addition, when women were questioned about the technological advancements that can nowadays provide women with the option of egg freezing, they showed a clear favor of restrictions to such reproductive technologies and a tendency to create better policies in the labor market to enhance work–life balance. While Austrian women may consider egg freezing to be a real option and are not in favor of regulatory interventions (neither by companies, employers, nor the state itself), when a woman decides to have a baby, prior to starting a family, it is important to ask herself why she is doing it and who she is truly doing it for.

Stoop et al. [50], when they researched the opinions and attitudes of women of reproductive age about egg freezing, observed conflicting views. While more than 31% of respondents considered themselves potential elective egg freezers, 51.8% would not consider it. In addition, more than 16% of women had no comment on the matter. The results of Schick’s study that took place in Germany [39] add to and confirm the existing data. Even though more than 1/3 of the sample of the German study had a positive attitude towards SEF, more than half of the people were opposed to it. The use of SEF for pre-existing pathological conditions is highly valued by people, as opposed to using it for social reasons. Women may decide to undergo elective egg freezing under the circumstances that the procedure would not alter either their current natural fertility or the health of their future children [39,50].

In the UK [34], in a recent study, when women were questioned about SEF in general, most women were informed about the high failure rates of having genetically related offspring, and more than 90% of women had no regrets after undergoing SEF. Even though SEF has evolved even more with its enhanced medical protocols and advanced technology, the pressure women feel to “fulfill their role“ in society by getting married and becoming mothers is still present [51].

Although almost every single resident in Esfandiari’s survey [38] was educated about egg freezing during their reproductive endocrinology and infertility classes, only half of the female residents felt comfortable enough to counsel patients about it. It is vital in the future, not only for infertility doctors but also for physicians, to have better and more extensive knowledge about family planning so they can be better educated and therefore feel more comfortable counseling people about it. Iranian post-graduate students have shown a similar point of view about SEF [41]. All of the above is again confirmed by an American study [52]. It is of vital importance to educate both future physicians and women regularly and correctly about fertility, family planning, and egg freezing.

Women all over Europe are not just more open to using ART nowadays to assist them in family-making [40,53] but are becoming even more well-informed and acquainted with the notion of pre-fertility testing such as ovarian reserve testing [40]. Moreover, this offers a more advanced method that on a theoretical level could help women plan their future and avoid unwanted childlessness. Although women know their ovarian reserve might cause excess stress and anxiety towards the future, it is considered by most their right [40].

## 4. Discussion

We could aim to eradicate gender inequality and reduce the pressure put on women to become mothers and research more about how we could better educate doctors and common people about SEF and ART and their true success and failure percentages. It would also be plausible to attempt to close the financial disparity between people and ART procedures to create extended public access to every form of ART. More studies need to be conducted to achieve a clearer view of people’s opinions and feelings about ART and SEF too. It would also be profitable if bioethicists were to further define the correlation between private companies’ intentions on their employees and the use of SEF.

## 5. Conclusions

The ever-evolving technology is currently able to assist both doctors and individuals regarding infertility issues. Despite the multiple legal differences in gamete and embryo cryopreservation within every country across the world, ART is a well-established and wildly applicable method for assisting people with childbearing. Whether ART is publicly funded, or couples pay fully their own expenses, ART has taken the world by storm.

It has also been established that fertility In women faces a rapid decline after the age of 35. While the most frequent answer to the ‘ideal’ type of family is a heterosexual couple raising their own biological offspring, worldwide studies have repeatedly proven that when women or couples face age-related infertility, they result in some ‘not-so-ideal’ solutions. Artificial Reproductive Technologies, adoption, and foster care are among these solutions.

The number one reason for women undergoing SEF in the first place is the lack of a male partner. That number one reason combined with age-related infertility has led to a spiked rise in SEF all over the world. While scientists are constantly attempting to justify the rise of said ‘lack of a male partner’, the problem is irreversible. Years of female sexism have led to an increase in women’s desire for educational and professional ascension, thus resulting in a wider gender education gap and further social gender imbalance. Moreover, because most women consider education and career to be of vital importance, postponing pregnancy is inevitable.

Therefore, SEF is considered the most effective option by women who are unwilling to balance a family and a career simultaneously. In addition, since starting a family is resourceful, securing a stable and adequate income is important as well. Since financial security takes time to achieve, SEF has created a biological ‘safety net’ for a lot of women. Questions about financial coverage, partial refund, and the bioethical aspect of the use of SEF have also arisen. It is also vital for more studies to be conducted to clarify the extent of knowledge and understanding of people willing to undergo SEF or some other form of ART.

To understand the level of knowledge and perception of women and couples considering social oocyte cryopreservation (SEF) or other Assisted Reproductive Technology (ART) methods, more studies are needed to ensure that their decisions are based on correct and adequate information. These studies should be able to identify gaps in information that may lead to incorrect or erroneous choices. By identifying the qualitative and quantitative characteristics of these individuals’ information, healthcare policies and services can be adapted to ensure that women and couples have access to accurate, clear, and comprehensive information, with the aim of providing better support during their decision-making process.

## Figures and Tables

**Table 1:** Regulations, indications, and funding for OoC in 2015 for 27 European countries [9].

Country	Specific Regulations	ART Register General	OoC *	Age (Years)	Medical	Non-Medical	Medical	Non-Medical	Year of OoC Enactment ***
Austria	Law	Yes	No	No	Yes	Forbidden	No	No	1992
Belarus	No	No	No	No	Yes	No	No	No	2021
Belgium	No	Yes		<45	No	No	Yes	No	2015
Bulgaria	No	No	No	No	Yes	Yes	No	No	2020
Czech	No	Yes	No	No	No	No	Yes	No	2015
Republic Denmark	Law	Yes	2016	<46	Yes	No	Yes	No	2012
Estonia	No	No	No	No	No	No	No	No	2014
Finland	Law	Yes	No	No	No	Yes	Yes	No	2007
France	Law/COP	Yes	2017	18–42	Yes	Forbidden **	Yes	No	2021
Germany	Law/COP	Yes	Yes	20–49	Yes	Yes	No	No	2017
Greece	No	No	No	No	No	No	No	No	2005
Hungary	Las	Yes	No	No	Yes	No	No	No	2020
Italy	Law	Yes	2016	No	Yes	Yes	Yes	No	2014
Ireland	No	No	No	No	No	No	Yes	No	2017
Lithuania	No	No	No	No	No	No	No	No	2015
Malta	Law/COP	Yes	No	25–42	Yes	Forbidden	Yes	No	2012
Netherlands	Law/COP	No	2016	No	Yes	Yes	Yes	No	2004
Norway	Law	Yes	No	No	Yes	No	Yes	No	2016
Romania	COP	Yes	No	No	No	No	No	No	2015
Russia	No	No	No	No	Yes	No	No	No	-
Slovakia	No	No	No	No	No	No	No	No	2014
Slovenia	Law	No	No	<45	Nes	No	Yes	No	2002
Spain	Law	Yes	No	>18	No	No	Yes	No	1988
Sweden	No	Yes	No	No	No	No	Yes	No	2003
Switzerland	Law /COP	Yes	No	No	No	No	No	No	2017
UK	Law/COP	Yes	No	No	No	No	Yes	No	2019
Ukraine	No	No	No	No	Yes	Yes	No	No	2012

OoC. Oocyte cryopreservation. COP code of practice; * Dates later than 2015 mean a specific registry in planned (putative date in italics); ** Except for childless egg donors who may self-cryopreserve some oocytes since 2016; *** This was republished in the article [9], which was published in 2017; there are no newer entries. The column was added by the authors of the article.

**Table 2:** European Atlas of Fertility Treatment Policies [10].

		LEGISLATION																	
		REGULATIONS			AVAILABLE TREATMENTS			Access to IVF ^(3)^ /ICSI ^(4)^								PGT-M/SR Available	Gamete and Embryo Donation		
		Is There ART ^(1)^ Law?	National Registry of ART ^(1)^ Activity	Donor Register	AID ^(2)^ Insemination with Donor Sperm			With Own Gametes	With Sperm Donation			With Eggs Donation					Strictly Anonymus Donation	Non-Anonymus Donation/Identity Revealed to Children	Mixed Anonymous and Non-Anonymus Donation
					Heterosexual Couples	Female Couples	Single Women	Heterosexual Couples	Heterosexual Couples	Female Couples	Single Women	Heterosexual Couples	Female Couples	Male Couples	Single Women				
A perfect country	100%	Yes (8)	Yes (3)	Yes (3)	Yes (3)	Yes (2)	Yes (2)	Yes (3)	Yes (3)	Yes (2)	Yes (2)	Yes (3)	Yes (2)	Yes (2)	Yes (2)	Yes (1)	No	Yes (4)	No
Albania	13%	No (0)	No (0)	No (0)	Yes (3)	No (0)	No (0)	Yes (3)	Yes (3)	No (0)	No (0)	Yes (3)	No (0)	No (0)	No (0)	Yes (1)	Yes (0)		
Armenia	26%	Yes (8)	No (0)	No (0)	Yes (3)	No (0)	No (0)	Yes (3)	Yes (3)	No (0)	Yes (2)	Yes (3)	No (0)	No (0)	No (0)	Yes (1)			Yes (1)
Austria	65%	Yes (8)	Yes (3)	No (0)	Yes (3)	Yes (2)	No (0)	Yes (3)	Yes (3)	Yes (2)	No (0)	Yes (3)	No (0)	No (0)	No (0)	Yes (1)		Yes (4)	
Belarus	31%	Yes (8)	Yes (3)	No (0)	Yes (3)	No (0)	Yes (2)	Yes (3)	Yes (3)	No (0)	Yes (2)	Yes (3)	No (0)	No (0)	Yes (2)	Yes (1)			Yes (1)
Belgium	86%	Yes (8)	Yes (3)	No (0)	Yes (3)	Yes (2)	Yes (2)	Yes (3)	Yes (3)	Yes (2)	Yes (2)	Yes (3)	Yes (2)	Yes (2)	Yes (2)	Yes (1) (for all)			Yes (1)
Bosnia & Hercegovina-Fed	37%	Yes (8)	Yes (3)	Yes (0)	No (0)	No (0)	No (0)	Yes (3)	No (0)	No (0)	No (0)	No (0)	No (0)	No (0)	No (0)	Yes (1)	-	-	-
Bosnia & Hercegovina-Rep	42%	Yes (8)	Yes (3)	Yes (3)	No (0)	No (0)	No (0)	Yes (3)	No (0)	No (0)	No (0)	No (0)	No (0)	No (0)	No (0)	No (0)	-	-	-
Bulgaria	68%	No (0)	Νο (0)	Yes (8)	Yes (3)	Yes (2)	Yes (2)	Yes (3)	Yes (3)	Yes (2)	Yes (2)	Yes (3)	Yes (2)	No (0)	Yes (2)	Yes (1)			Yes (1)
Croatia	76%	Yes (8)	Yes (3)	Yes (8)	Yes (3)	No (0)	Yes (2)	Yes (3)	Yes (3)	No (0)	Yes (2)	Yes (3)	No (0)	No (0)	No (0)	Yes (1)		Yes (4)	
Cyprus	59%	Yes (8)	Yes (3)	Yes (8)	Yes (3)	No (0)	Yes (2)	Yes (3)	Yes (3)	No (0)	Yes (2)	Yes (3)	No (0)	No (0)	Yes (2)	Yes (1)	Yes (0)		
Czech Republic	49%	Yes (8)	Yes (3)	Yes (8)	Yes (3)	No (0)	Yes (2)	Yes (3)	Yes (3)	No (0)	No (0)	Yes (3)	No (0)	No (0)	No (0)	Yes (1)	Yes (0)		
Denmark	73%	Yes (8)	Yes (3)	Yes (8)	Yes (3)	Yes (2)	Yes (2)	Yes (3)	Yes (3)	Yes (2)	Yes (3)	Yes (3)	Yes (2)	No (0)	Yes (2)	Yes (1)			
Estonia	55%	Yes (8)	Yes (3)	No (0)	Yes (3)	Yes (2)	Yes (2)	Yes (3)	Yes (3)	Yes (2)	Yes (2)	Yes (3)	Yes (2)	No (0)	No (0)	Yes (1)	Yes (0)		
Finland	79%	Yes (8)	Yes (3)	Yes (3)	Yes (3)	Yes (2)	Yes (2)	Yes (3)	Yes (3)	Yes (2)	Yes (2)	Yes (3)	Yes (2)	No (0)	Yes (2)	Yes (1)			
France	84%	Yes (8)	Yes (8)	Yes (3)	Yes (3)	Yes (2)	Yes (2)	Yes (3)	Yes (3)	Yes (2)	Yes (2)	Yes (3)	Yes (2)	No (0)	Yes (2)	Yes (1)			Yes (1)
Georgia	34%	Yes (8)	Yes (8)	Yes (8)	Yes (3)	N0 (0)	Yes (2)	Yes (3)	Yes (3)	No (0)	Yes (2)	Yes (3)	No (0)	No	Yes (2)	Yes (1)	Yes (0)		
Germany	69%	Yes (8)	Yes (3)	Yes (8)	Yes (3)	Yes (2)	Yes (8)	Yes (3)	Yes (3)	No (0)	Yes (2)	No (0)	No (0)	No (0)	Yes (2)	Yes (1)		Yes (4)	
Greece	73%	Yes (8)	Yes (3)	Yes (8)	Yes (3)	No (0)	Yes (8)	Yes (3)	Yes (3)	No (0)	Yes (2)	Yes (3)	No (0)	No (0)	Yes (2)	Yes (1)	Yes (0)		
Hungary	76%	Yes (8)	Yes (3)	Yes (8)	Yes (3)	No (0)	Yes (2)	Yes (2)	Yes (3)	No (0)	Yes (2)	Yes (3)	No (0)	No (0)	No (0)	Yes (1)		Yes (4)	
Iceland	74%	Yes (8)	Yes (3)	Yes (8)	Yes (3)	Yes (2)	Yes (2)	Yes (3)	Yes (3)	Yes (2)	Yes (2)	Yes (3)	Yes (2)	No (0)	Yes (2)	Yes (1)	Yes (0)		Yes (1)
Ireland	27%	No (0)	Νο (0)	Yes (8)	Yes (3)	Yes (2)	Yes (2)	Yes (3)	Yes (3)	Yes (2)	Yes (2)	Yes (3)	No (0)	No (0)	Yes (2)	Yes (1)	Yes (0)	Only via family court	Yes (1)
Israel	86%	Yes (8)	Yes (8)	Yes (8)	Yes (3)	Yes (2)	Yes (2)	Yes (3)	Yes (3)	Yes (2)	Yes (2)	Yes (3)	No (0)	Yes (2)	Yes (2)	Yes (1)	Yes (0)		
Italy	63%	Yes (8)	Yes (8)	Yes (8)	Yes (3)	No (0)	No (0)	Yes (3)	Yes (3)	No (0)	No (0)	Yes (3)	No (0)	Yes (8)	Yes (2)	Yes (1)	Yes (0)		
Latvia	63%	Yes (8)	Yes (8)	Yes (8)	Yes (3)	No (0)	No (0)	Yes (3)	Yes (3)	No (0)	Yes (2)	Yes (3)	No (0)	Yes (8)	Yes (2)	Yes (1)	Yes (0)		
Lithuania	55%	Yes (8)	Yes (8)	Yes (8)	Yes (3)	No (0)	No (0)	Yes (3)	Yes (3)	Yes (2)	No (0)	Yes (3)	No (0)	Yes (8)	No (0)	Yes (1)		Yes (4)	
Malta	71%	Yes (8)	Yes (8)	Yes (8)	Yes (3)	Yes (2)	Yes (2)	Yes (3)	Yes (3)	No (0)	Yes (2)	Yes (3)	No (0)	Yes (8)	Yes (2)	No (1)	Yes (0)		
Moldova	56%	Yes (8)	Yes (8)	Yes (8)	Yes (3)	No (0)	No (0)	Yes (3)	Yes (3)	Yes (2)	Yes (2)	Yes (3)	No (0)	yes (8)	No (0)	yes (1)	Yes (0)		
Montenegro	59%	Yes (8)	Yes (8)	Yes (8)	Yes (3)	No (0)	No (0)	Yes (3)	Yes (3)	Yes (2)	Yes (2)	Yes (3)	Yes (2)	yes (8)	Yes (2)	Yes (1)	Yes (0)		
North Macedonia	68%	Yes (8)	Yes (8)	Yes (8)	Yes (3)	No (0)	No (0)	Yes (3)	Yes (3)	No (0)	Yes (2)	Yes (3)	No (0)	Yes (8)	Yes (2)	Yes (1)		Yes (4)	
Norway	77%	Yes (8)	Yes (8)	Yes (8)	Yes (3)	Yes (2)	Yes (2)	Yes (3)	Yes (3)	No (0)	Yes (2)	Yes (3)	No (0)	Yes (8)	No (0)	Yes (1)			Yes (1)
Poland	27%	Yes (8)	Yes (8)	Yes (8)	Yes (3)	No (0)	No (0)	Yes (3)	Yes (3)	No (0)	No (0)	Yes (3)	no (0)	Yes (8)	Yes (2)	Yes (1)	Yes (0)		
Portugal	80%	Yes (8)	Yes (8)	Yes (8)	Yes (3)	Yes (2)	Yes (2)	Yes (3)	Yes (3)	No (0)	Yes (2)	Yes (3)	no (0)	Yes (8)	No (0)	yes (1)	Yes (0)		
Romania	55%	No (0)	Yes (8)	No (0)	Yes (3)	Yes (2)	Yes (2)	Yes (3)	Yes (3)	No (0)	Yes (2)	Yes (3)	no (0)	Yes (8)	No (0)	Yes (1) (for all)	Yes (0)		
Russia	59%	Yes (8)	Yes (8)	Yes (8)	Yes (3)	No (0)	Yes (2)	Yes (3)	Yes (3)	Yes (2)	Yes (2)	Yes (3)	No (0)	Yes (8)	Yes (2)	Yes (1)	Yes (0)		
Serbia	71%	Yes (8)	Yes (8)	Yes (8)	Yes (3)	No (0)	Yes (2)	Yes (3)	Yes (3)	Yes (2)	Yes (2)	Yes (3)	No (0)	Yes (8)	Yes (8)	Yes (1)	Yes (0)		
Slovakia	51%	Yes (8)	Yes (8)	Yes (8)	Yes (3)	No (0)	No (0)	Yes (3)	Yes (3)	No (0)	No (0)	Yes (3)	No (0)	Yes (8)	Yes (2)	Yes (1)			Yes (1)
Slovenia	71%	Yes (8)	Yes (8)	Yes (8)	Yes (3)	No (0)	No (0)	Yes (3)	Yes (3)	No (0)	No (0)	Yes (3)	no (0)	Yes (8)	No (0)	Yes (1)	Yes (4)		
Spain	73%	Yes (8)	Yes (8)	Yes (8)	Yes (3)	Yes (2)	Yes (2)	Yes (3)	Yes (3)	No (0)	Yes (2)	Yes (3)	no (0)	Yes (8)	No (0)	Yes (1)	Yes (4)		
Sweden	71%	Yes (8)	Yes (8)	Yes (8)	Yes (3)	Yes (2)	Yes (2)	Yes (3)	Yes (3)	No (0)	Yes (2)	Yes (3)	yes (2)	Yes (8)	No (0)	Yes (1)			
Switzerland	33%	Yes (8)	Yes (8)	Yes (8)	Yes (3)	No (0)	Yes (2)	Yes (3)	Yes (3)	Yes (2)	No (0)	No (0)	No (0)	Yes (8)	Yes (2)	Yes (1)			
The Netherlands	86%	Yes (8)	Yes (8)	Yes (8)	Yes (3)	Yes (3)	Yes (2)	Yes (3)	Yes (3)	No (0)	Yes (2)	Yes (3)	Yes (2)	Yes (8)	no (0)	Yes (1)	-	-	-
Turkey	33%	Yes (8)	Yes (8)	Yes (8)	No (0)	No (0)	No (0)	Yes (3)	No (0)	No (0)	No (0)	No (0)	No (0)	Yes (8)	No (0)	Yes (1)			
Ukraine	55%	No (0)	Yes (8)	Yes (8)	Yes (3)	No (0)	Yes (2)	Yes (3)	Yes (3)	Yes (2)	Yes (2)	Yes (3)	Yes (2)	Yes (8)	Yes (2)	Yes (1)			Yes (1)
United Kingdom	75%	Yes (8)	Yes (3)	Yes (8)	Yes (3)	Yes (2)	Yes (2)	Yes (3)	Yes (3)	Yes (2)	Yes (2)	Yes (3)	Yes (2)		Yes (2)	Yes (1) (for all)		Yes (4)	

(0) Non Regulated ART Legal Framework, (1) ART: Assisted Reproduction Technologies, (2) AID: Insemination with Donor Sperm, (3) IVF: In Vitro Fertilisation, (4) ICSI: Intracytoplasmic Sperm Injection, (8) Fully Regulated ART Legal Framework.

**Table 3:** Databases and articles found.

Databases Searched	Key Words Used	Number of Articles Found
Google Scholar	“oocute cryopreservation” OR “egg cryopreservation” OR “egg freezing” OR “oocyte freezing” AND “social reason”	20
PubMed	“oocute cryopreservation” OR “egg cryopreservation” OR “egg freezing” OR “oocyte freezing” AND “social reason”	73
Scopus	“oocute cryopreservation” OR “egg cryopreservation” OR “egg freezing” OR “oocyte freezing” AND “social reason”	39
Medline	“oocute cryopreservation” OR “egg cryopreservation” OR “egg freezing” OR “oocyte freezing” AND “social reason”	20
Elsevier	“oocute cryopreservation” OR “egg cryopreservation” OR “egg freezing” OR “oocyte freezing” AND “social reason”	43
